# Supplementary material for: Collaborative intelligence in AI: Evaluating the performance of a council of AIs on the USMLE
Source: PLOS Digit Health. 2025 Oct 9;4(10):e0000787. doi: 10.1371/journal.pdig.0000787 (PMC12510544; doi:10.1371/journal.pdig.0000787)
Supplement: S2 File — (ZIP) [file pdig.0000787.s002.zip › S2 Data Raw data files containing unprocessed question inputs and LLM outputs including transcripts of deliberations/STEP3_Question-33-1_Type-MC-NJ.docx]

**Facilitator** **[Round 1]:** A male neonate, who was born at 36 weeks' gestation 2 hours ago in the labor and delivery unit of the hospital, now shows signs of respiratory difficulty. The mother, an 18-year-old primigravid woman, smoked one pack of cigarettes daily throughout her pregnancy. She received prenatal care during most of the pregnancy. One episode of chlamydial cervicitis was detected during the last trimester and treated with azithromycin. The neonate was born via cesarean delivery due to fetal heart rate decelerations. The amniotic fluid was stained with light particulate meconium. Apgar scores were 9 and 9 at 1 and 5 minutes, respectively. The patient is 50 cm (20 in; 50th percentile) long and weighs 3005 g (6 lb 10 oz; 50th percentile); head circumference is 35 cm (14 in; 50th percentile). The infant's vital signs now are temperature 36.6°C (97.8°F), pulse 150/min, and respirations 70/min. Pulse oximetry on room air shows an oxygen saturation of 95%. Physical examination discloses mild subcostal and intercostal retractions. Chest x-ray shows prominent pulmonary vascular markings and fluid in the intralobar fissures. Which of the following diagnoses is most likely and explain your rationale on each choice? (A) Chlamydial pneumonia (B) Group B streptococcal sepsis (C) Meconium aspiration syndrome (D) Respiratory distress syndrome (E) Transient tachypnea of newborn

**AI_A:** RESTATING THE QUESTION: The recent neonatal case in question involves a full-term male presenting with respiratory difficulty, associated with certain pre-existing maternal conditions like her smoking habits during pregnancy and a treated instance of chlamydial cervicitis. Concurrently, we provided with certain observations made on the neonate post-birth (meconium-stained amniotic fluid, chest X-ray findings indicating fluid in intralobar fissures, and respective Apgar scores). The task at hand is to determine the likely cause of the baby's current respiratory signs. PLANNING: The possible options each represent distinct pathologies. Chlamydial pneumonia is respiratory disease induced by Chlamydia trachomatis, often manifesting between 2-19 weeks post-partum. Group B streptococcal sepsis is an early-onset, severe bacterial infection primarily contracted perinatally from maternal colonization, having onset throughout the first week of life. Meconium aspiration syndrome occurs when the baby inhales a mixture of meconium and amniotic fluid. Respiratory distress syndrome relates to lack of surfactant causing difficulty in breathing, while Transient Tachypnea of the Newborn (TTN) is a benign, temporary breathlessness following delivery, often resulting from delayed clearance of lung fluid. CLINICAL REASONING: In this case, considering the neonate's clinical features and given the delay in onset of presentation (2 hours post-delivery), early chlamydial pneumonia and Group B streptococcal sepsis are unlikely as they typically manifest later in infancy and within first week of life respectively. Meconium aspiration syndrome usually presents in babies with pre-existent intrauterine stress, which induces the passage of meconium, but the degree of respiratory difficulty doesn't seem severe enough in this baby. The baby's adequate Apgar score and overall vitality further decrease the probability of respiratory distress syndrome, which is also more common in premature infants. CONCLUDING: Adding all these factors together leans us towards Transient Tachypnea of the Newborn (TTN) as the probable diagnosis. This condition is widely present in neonates delivered via cesarean section, as in this case. TTN is usually marked by tachypnea manifesting within first few hours of life, often supplemented by other symptoms like mild intercostal or subcostal retractions, consistent with the neonate's presentation. RESPONSE: Given the above information and rationalization, it seems most plausible that this neonate is suffering from Transient Tachypnea of the Newborn (Option E).

**AI_C:** RESTATING: A 36-week old neonate male is showing signs of respiratory distress, including rapid breathing and mild subcostal and intercostal retractions. The mother was a heavy smoker throughout pregnancy, and had an incident of chlamydial cervicitis during the third trimester, but was treated with azithromycin. The delivery was a cesarean due to deceleration of the fetal heart rate, and the amniotic fluid had light meconium. The baby's vital signs show elevated pulse and breathing rates, with chest x-ray showing evidence of fluid in the intralobar fissures. The baby's measurements are within the 50th percentile for his age. We are asked to identify the most likely diagnosis based on the given choices and discuss our reasoning for each. PLANNING: On examination, the baby's symptoms are primarily respiratory related. However, differential diagnosis could include both infectious and non-infectious conditions. The choices provided include chlamydial pneumonia (from maternal infection during pregnancy), Group B streptococcal sepsis (from vertical transmission during birth), Meconium Aspiration Syndrome (from in utero meconium release), Respiratory Distress Syndrome (from premature birth), and Transient Tachypnea of the Newborn (delayed clearance of fetal lung fluid). CLINICAL REASONING: Chlamydial pneumonia typically presents one to three weeks after birth and is marked by a staccato cough, unlike the symptoms exhibited. Group B streptococcal sepsis usually manifest with a high fever, lethargy, poor feeding, and respiratory distress, while lacking the maternal risk and the baby's current symptoms. Meconium Aspiration Syndrome would likely show up as coarse crackles, wheezing, and cyanosis, and though this baby has mild respiratory distress, there are no signs of cyanosis. Also, the light meconium staining doesn't necessarily indicate aspiration. Respiratory Distress Syndrome symptom onset typically occurs immediately after birth and worsens over the first 72 hours, but the baby's symptoms do not align with the typical presentation. CONCLUDING: The neonate presented with mild subcostal and intercostal retractions, a rapid respiratory rate, and an x-ray showing both prominent pulmonary vascular markings and fluid in the intralobar fissures. These clinical findings align most with Transient Tachypnea of the Newborn (TTN), which is often seen in infants born through cesarean delivery, as this can delay the absorption and clearance of fetal lung fluid. RESPONSE: Based on the clinical picture, the diagnosis of Transient Tachypnea of the Newborn (E) is most probable. The symptoms match the clinical signs of TTN, and the neonate's mode of delivery further supports this diagnosis. Other options are less likely based on onset, symptom presentation, and risk factors.

**AI_B:** 1) RESTATING THE QUESTION: The question involves a newborn baby boy who is showing signs of trouble with breathing. He was born almost at the full term (36 weeks) through a surgical procedure, the C-section, because of a drop in his heart rate. The mother is a teenager with a smoking habit and also had an episode of a sexually transmitted infection (chlamydial cervicitis) during her last pregnancy term, which was appropriately treated. Furthermore, the baby's first bowel movement (meconium) was noted in the amniotic fluid during birth. Despite this, the infant's physical measurements and Apgar scores (used to score the physical health of newborns) are normal. The current symptoms being displayed by the baby are retraction (a sign of working hard to breathe), an escalated heartbeat and rapid breathing. The child's x-ray has revealed an increase in lung blood vessels and fluid congestion. We need to analyze the potential diagnoses related to these symptoms. Those options are Chlamydial pneumonia, Group B streptococcal sepsis, Meconium aspiration syndrome, Respiratory distress syndrome, and Transient tachypnea of the newborn. 2) PLANNING: The child has prominent respiratory symptoms such as retractions and tachypnea. These could be caused by numerous conditions, generally falling into categories like lung infections, aspiration of meconium, immature lungs, or temporary issues related to fluid clearance. We need to rationalize each option. (A) possibilities of a lung infection caused by Chlamydia as the mother had an infection during her pregnancy. (B) A systemic infection triggered by the Group B Streptococcus bacteria. (C) A condition caused by the baby inhaling his first bowel content (meconium) into his lungs. (D) A lung condition due to premature birth. (E) A temporary breathing issue due to extra fluid in the baby's lungs. 3) CLINICAL REASONING: Correlating with each diagnosis: (A) The mother had a Chlamydia infection, but it was treated. Even if untreated, Chlamydial Pneumonia tends to present after a few weeks of life, and often not as severely. (B) Group B streptococcal sepsis usually presents with more systemic symptoms and not prominent lung findings. (C) Meconium Aspiration Syndrome commonly shows coarse and patchy infiltrates on chest x-ray, not typically seen here. (D) Respiratory Distress Syndrome, common in premature infants, usually presents immediately post-birth. The x-ray often shows low lung volume and a ground-glass appearance, not seen here. (E) Transient Tachypnea of The Newborn (TTN) often occurs in neonates born via cesarean section with no labor due to inadequate clearance of lung fluid. Chest x-ray typically shows hyperinflation and fluid in the fissures, aligning with this case's presentation. 4) CONCLUDING: Based on the temporal presentation, absence of more systemic symptoms or specific radiographic signs, and the context of a cesarean delivery, the most likely diagnosis becomes TTN. 5) RESPONSE: Therefore, the most likely diagnosis for this baby is (E) Transient tachypnea of the newborn, a condition where the newborn has temporary trouble clearing the lung fluid and breathes rapidly for a short period. This diagnosis connects to the fact that she was born via cesarean delivery and the chest x-ray findings are congruent with this condition.

**AI_D:** RESTATING THE QUESTION: The clinical situation presented discusses a newborn male who exhibits respiratory difficulty and certain findings in his chest x-ray. We are asked to pick the most likely diagnosis from Chlamydial pneumonia, Group B streptococcal sepsis, Meconium aspiration syndrome, Respiratory distress syndrome, and Transient tachypnea of the newborn. PLANNING: The uncertainty arises from the multiple possible causes of the neonate's presentation. Some important background factors to consider include the mother's smoking habit, her chlamydial cervicitis, the neonate's delivery by cesarean section, and the presence of light particulate meconium in the amniotic fluid. A common feature with all the presented conditions is respiratory difficulty, hence the importance of looking critically at other distinctive features. (1) Chlamydial Pneumonia: typically develops 2-3 weeks after birth and would not present immediately after delivery. (2) Group B streptococcal sepsis: Group B streptococcus infection is likely to present with more severe symptoms like temperature instability, lethargy, poor feeding, or apnea. (3) Meconium Aspiration Syndrome: This should be anticipated in neonates born through meconium-stained amniotic fluid but the chest x-ray findings typically show areas of overdistension and atelectasis forming a 'patchy' pattern. (4) Respiratory Distress Syndrome: This usually affects premature neonates and presents within minutes after birth. The chest x-ray shows a 'ground-glass' appearance due to atelectasis. (5) Transient Tachypnea of Newborn (TTN): This condition may present with signs of respiratory distress such as fast breathing within hours after birth. Chest x-ray shows hyperinflation of the lungs and fluid in the fissures. CLINICAL REASONING: In the present case, the neonate showed signs of respiratory difficulty 2 hours after birth and had a chest x-ray showing prominent pulmonary vascular markings and fluid in the interlobar fissures. The mother smoked cigarettes throughout her pregnancy, but no indications of any nicotine effect on the fetus were provided such as growth retardation. Although the mother had chlamydial cervicitis in the last trimester, it was treated and chlamydial pneumonia would not present so soon after birth. No severe symptoms were reported that might suggest a severe infection like Group B streptococcal sepsis. Despite the presence of light particulate meconium in the amniotic fluid, the findings in the chest x-ray were not typical for Meconium Aspiration Syndrome. The baby was born at 36 weeks which puts him nearly to term, minimizing the likelihood of respiratory distress syndrome, besides the presentation does not match. CONCLUDING: Since none of the alternate assumptions provide a fitting explanation for the neonate's presentation, we may logically infer that the neonate's respiratory distress could be due to Transient Tachypnea of the Newborn. TTN usually presents within the first few hours after birth, which is compatible with this case. Chest x-ray showing prominent pulmonary vascular markings and fluid in the fissures are typical findings in TTN, supporting this diagnosis. The cause of TTN is thought to be delayed absorption of fetal lung fluid resulting from delivery by caesarean section without labour or rapid delivery, conditions that match this scenario. RESPONSE: Given the clinical presentation, timeline of events, and chest x-ray findings, the most likely diagnosis for this neonate's respiratory difficulty is Transient Tachypnea of Newborn (E). The other options would not present as described in the scenario or don't match the timeline and/or other clinical findings.

**AI_E:** RESTATING THE QUESTION: This recently born male neonate, born at 36 weeks' gestation or slightly premature, is showing symptoms of respiratory distress such as rapid breathing and mild intercostal and subcostal retractions. Noteworthy are facts that the mother smoked regularly throughout the pregnancy, was treated for chlamydial cervicitis and that the amniotic fluid had light meconium. We are asked to use these clinical findings to determine the most likely diagnosis from a given list of conditions: Chlamydial pneumonia, Group B streptococcal sepsis, Meconium aspiration syndrome, Respiratory distress syndrome and Transient tachypnea of newborn. PLANNING: Each condition has distinguishing features that can help direct the diagnosis, assuming they are present. (A) Chlamydial pneumonia often presents later, 2-12 weeks after birth, with conjunctivitis followed by a staccato cough in an afebrile infant. (B) Group B streptococcal sepsis would typically present with a broad range of systemic signs like lethargy, poor feeding, temperature instability and in some severe cases, respiratory distress. (C) Meconium aspiration syndrome might present within a few hours after birth, with severe respiratory distress including signs like greenish staining of the skin, nails and umbilical cord, and, often, cyanosis or bluish coloration of skin due to low oxygen. (D) Respiratory distress syndrome could present immediately after birth, mainly in preterm infants, with rapid, shallow breathing and retractions. Chest X-ray typically reveals a "ground-glass" appearance. (E) Transient tachypnea of newborn usually occurs in babies born via cesarean section, with respiratory distress presenting shortly after birth and typically resolves within 24-72 hours. Chest x-ray may show prominent vascular markings and fluid in the fissures. CLINICAL REASONING: Considering the clinical findings in the presented case, the neonate shows rapid breathing and mild retractions as the signs of respiratory distress, which are common in many neonatal respiratory conditions. The mother's chlamydial cervicitis and smoking habit during pregnancy, although both concerning, are not directly indicative of the conditions being evaluated. The presence of light meconium in the amniotic fluid could contribute to Meconium aspiration syndrome, but other symptoms like greenish staining and severe respiratory distress are not reported. C-section can delay absorption of lung fluid, causing what is known as Transient tachypnea of newborn (TTN). The chest x-ray finding showing prominent vascular marking and fluid in the fissures is typical for TTN, not for other conditions like chlamydial pneumonia or Group B streptococcal sepsis. The fact that respiratory distress started few hours after birth also points against Respiratory distress syndrome, which usually presents right after birth. CONCLUDING: Therefore, taking into consideration all provided clinical data and reasoning through each potential diagnosis, the most probable condition for this neonate's respiratory distress would be Transient tachypnea of newborn. RESPONSE: The newborn's condition is most likely (E) Transient tachypnea of newborn, a condition due to delayed absorption of fetal lung fluid and characteristically presents with tachypnea and respiratory distress within the first several hours of life. The chest x-ray findings of prominent vascular markings and fluid in the intralobar fissures are also consistent with this condition. Other conditions from the options do not align well with the complete set of clinical data provided.

**Facilitator** The consensus among the AI responses is that the most likely diagnosis for the newborn male's respiratory difficulty is Transient Tachypnea of the Newborn (TTN). This conclusion is based on several factors: 1. The neonate's symptoms, including rapid breathing and mild subcostal and intercostal retractions, are consistent with TTN. These symptoms typically present within the first few hours after birth, which aligns with the timeline in this case. 2. The chest x-ray findings of prominent pulmonary vascular markings and fluid in the intralobar fissures are typical for TTN. These findings suggest delayed absorption of fetal lung fluid, a common cause of TTN. 3. The neonate was delivered via cesarean section, a factor often associated with TTN. This is because cesarean delivery can delay the absorption and clearance of fetal lung fluid. 4. Other potential diagnoses were considered but deemed less likely based on the neonate's symptoms, the timeline of their onset, and other clinical findings. For example, Chlamydial pneumonia and Group B streptococcal sepsis typically present later and with different symptoms. Meconium Aspiration Syndrome would likely present with more severe respiratory distress and other symptoms not reported in this case. Respiratory Distress Syndrome usually affects premature neonates and presents immediately after birth, which does not align with this case. Therefore, given the clinical presentation, timeline of events, and chest x-ray findings, the consensus is that the neonate's respiratory difficulty is most likely due to Transient Tachypnea of Newborn.
